# Draft Genome Sequences of Two Fusarium oxysporum Isolates Cultured from Infected Zinnia hybrida Plants Grown on the International Space Station

**DOI:** 10.1128/genomeA.00326-18

**Published:** 2018-05-17

**Authors:** Camilla Urbaniak, Gioia Massa, Mary Hummerick, Christina Khodadad, Andrew Schuerger, Kasthuri Venkateswaran

**Affiliations:** a Jet Propulsion Laboratory, California Institute of Technology, Pasadena, California, USA; b Exploration Research and Technology Programs, NASA, Kennedy Space Center, Florida, USA; c AECOM, Kennedy Space Center, Florida, USA; d Space Life Sciences Lab, University of Florida, Gainesville, Florida, USA

## Abstract

Here, we present the whole-genome sequences of two Fusarium oxysporum isolates cultured from infected Zinnia hybrida plants that were grown onboard the International Space Station (ISS).

## GENOME ANNOUNCEMENT

The Fusarium oxysporum species complex represents one of the most important plant pathogens worldwide, causing disease in many economically important plants and crops (1), but can also cause opportunistic infections in humans (2, 3). However, F. oxysporum, like other fungi, produces many bioactive compounds that are beneficial to humans (4, 5) and could be exploited for use in the pharmaceutical/medical industries. During the course of the “Veggie” project (a vegetable production system flown onboard the International Space Station [ISS] to study the effects of the space environment on plant growth and function), Zinnia hybrida plants became afflicted with a foliar, stem, and root rot disease caused by the fungus Fusarium oxysporum. Isolates were cultured from the leaf (VEG-01C1) and the root (VEG-01C2) of these infected plants, and the draft whole-genome sequences are described herein.

The whole-genome sequences were paired-end sequenced (2 × 100 bp) on the Illumina HiSeq platform with a 350-bp insert size. A total of 33 million reads (GC content, 47.5%) and 46 million reads (GC content, 46.8%) were obtained from VEG-01C1 and VEG-01C2, respectively. Trimmomatic, on the Galaxy server (https://usegalaxy.org), was used to remove the sequencing adaptors (settings, max mismatch, 2; how accurate the match between the two adaptor ligated reads, 30; how accurate the match between any adaptor, 10) and to trim the leading and trailing ends (settings, minimum quality required to keep a base, 3). Postprocessed reads were *de novo* assembled with ABySS version 2.0.2 (6) using *k*-mer sizes of 80 (VEG-01C1) and 88 (VEG-01C2). The VEG-01C1 assembly resulted in a genome size of 49.3 Mb, with an *N*_50_ value of 376,797 bp. The number of scaffolds generated was 6,455, with a max scaffold length of 1,817,733 bp. The number of scaffolds over 1 kb was 588. The VEG-01C2 assembly resulted in a genome size of 48.9 Mb, with an *N*_50_ of 334,342 bp. The number of scaffolds generated was 6,398, with a max scaffold length of 2,326,957 bp. The number of scaffolds over 1 kb was 637.

The assembled genomes were compared to those of 66 F. oxysporum isolates downloaded from NCBI (ftp://ftp.ncbi.nlm.nih.gov/genomes/genbank/fungi/Fusarium_oxysporum/), as well as those of two isolates cultured from ISS environmental surfaces, using (i) 10 phylogenetically informative loci (7) and (ii) the presence/absence of effector proteins (8). Both methods showed that VEG-01C1 and VEG-01C2 were most closely related to F. oxysporum IMV-00293, an isolate cultured in the aftermath of the Chernobyl disaster (GenBank assembly accession number GCA_001931975). To note, this isolate was incorrectly identified and deposited as Fusarium solani (9). Interestingly, F. oxysporum strains VEG-01C1, VEG-01C2, and IMV-00293 (i.e., the Chernobyl strain) were very similar to the clinical F. oxysporum strain FOSC 3-a (GenBank assembly accession number GCA_000271745), an isolate cultured from blood from a patient in the United States suffering from fusariosis. Comparative genomics of these Z. hybrida leaf and root fungal strains with the Chernobyl (IMV-00293) strain could provide insight into which genes could allow for growth in extreme environments, such as those involved in radiation resistance.

### Accession number(s).

The assembled whole-genome sequences have been deposited in DDBL/EMBL/GenBank under the accession numbers PXUO00000000 (VEG-01C1) and PXUN00000000 (VEG-01C2). The strains have also been deposited in NASA’s GeneLab; https://genelab-data.ndc.nasa.gov/genelab/accession/GLDS-177/. These are the first versions.
